# Capybara Oil‐Based Nanoemulsion Attenuates Obesity‐Induced Renal Injury Through Modulation of Inflammation, Oxidative Stress, and Fibrosis in Mice

**DOI:** 10.1002/cbf.70316

**Published:** 2026-10-05

**Authors:** Priscila G. Pereira, Luciana L. Alves, Bianca T. Ciambarella, Stela R. Ferrarini, Ana Lúcia R. Nascimento, Cherley B. V. Andrade, Alan Cesar N. Moraes, Andressa Bernardi, Fernanda V. Guimarães, Miguel P. Campos, Denia M. S. Valladão, Letícia Cruz, Jemima F. R. da Silva, Kíssila Rabelo, Jorge J. de Carvalho

**Affiliations:** ^1^ Ultrastructure and Tissue Biology Laboratory, Department of Histology and Embryology, Institute of Biology Roberto Alcantara Gomes (IBRAG) Rio de Janeiro State University (UERJ) Rio de Janeiro Brazil; ^2^ Electron Microscopy Laboratory, Institute of Biology Roberto Alcantara Gomes (IBRAG) Rio de Janeiro State University (UERJ) Rio de Janeiro Brazil; ^3^ Graduate Program in Clinical and Experimental Physiopathology (FISCLINEX), Faculty of Medical Sciences Rio de Janeiro State University (UERJ) Rio de Janeiro Brazil; ^4^ Pharmaceutical Nanotechnology Laboratory Federal University of Mato Grosso (UFMT) Sinop Brazil; ^5^ Translational Endocrinology Laboratory, Carlos Chagas Filho Institute of Biophysics (IBCCF) Federal University of Rio de Janeiro (UFRJ) Rio de Janeiro Brazil; ^6^ Electron Microscopy Laboratory, Institute of Biology Fluminense Federal University (UFF) Niterói Brazil; ^7^ Laboratory of Inflammation, Oswaldo Cruz Institute (IOC) Oswaldo Cruz Foundation (Fiocruz) Rio de Janeiro Brazil; ^8^ Quality Control Laboratory Federal University of Mato Grosso (UFMT) Sinop Brazil; ^9^ Pharmaceutical Technology Laboratory, Department of Industrial Pharmacy Federal University of Santa Maria (UFSM) Santa Maria Brazil; ^10^ Graduate Program in Human Biology and Experimental Biology (BHEx), Institute of Biology Roberto Alcantara Gomes (IBRAG) Rio de Janeiro State University (UERJ) Rio de Janeiro Brazil; ^11^ Interdisciplinary Medical Research Laboratory, Oswaldo Cruz Institute (IOC) Oswaldo Cruz Foundation (Fiocruz) Rio de Janeiro Brazil

**Keywords:** capybara oil, kidney injury, nanoemulsion, obesity, obesity, omega‐3

## Abstract

Obesity is a multifactorial disorder associated with systemic metabolic alterations and increased risk of renal injury, largely driven by chronic inflammation and oxidative stress. Bioactive lipids, particularly polyunsaturated fatty acids, have been shown to exert protective effects; however, their instability and susceptibility to oxidation may limit their therapeutic potential. In this study, we investigated the effects of a capybara oil‐based nanoemulsion (NE‐CO) on renal dysfunction in a mouse model of obesity. Male mice were subjected to diet‐induced obesity and treated with NE‐CO (100 mg/kg/day) for 4 weeks. Metabolic parameters, systolic blood pressure, urinary biomarkers, and renal morphology were evaluated. In addition, markers of inflammation, oxidative stress, and fibrosis were assessed to elucidate potential mechanisms. NE‐CO treatment significantly reduced body mass and systolic blood pressure, while improving renal function, as evidenced by decreased proteinuria and normalization of urinary creatinine levels. Histological and ultrastructural analyzes revealed marked improvements in renal cortical organization. These effects were accompanied by reduced expression of pro‐inflammatory markers, enhanced antioxidant defenses, and attenuation of fibrotic remodeling. Collectively, these findings demonstrate that NE‐CO mitigates obesity‐induced renal injury, at least in part, through modulation of inflammatory, oxidative, and fibrotic pathways. This study highlights the therapeutic potential of bioactive lipid delivery systems in the context of metabolic and renal disorders.

## Significance Statement

Obesity can cause structural and functional changes in the kidneys through interconnected metabolic, inflammatory, and fibrotic processes. This study investigated whether a nanoemulsion containing capybara oil could attenuate renal injury in obese mice. Treatment with the capybara oil‐based nanoemulsion improved renal structure and function and modulated inflammatory and antioxidant responses, while reducing markers associated with renal fibrosis. These effects were more pronounced with the nanoemulsion than with free capybara oil. The findings provide evidence that nanoemulsification may enhance the biological effects of capybara oil and support further investigation of this formulation as a potential strategy for preventing or treating obesity‐associated kidney injury.

**Abbreviations**
BM: body mass
CAT: catalase
CKD: chronic kidney disease
CO: capybara oil
CON: capybara oil nanoemulsion
EMT: epithelial‐mesenchymal transition
FAs: fatty acids
HE: hematoxilin‐eosin
IL‐10: interleukin 10
LDL: low density lipoprotein
PAS: periodic acid‐schif
PSR: picro sirius red
PUFA: polyunsaturated fatty acid
SBP: systolic blood pressure
SOD: superoxide dismutase
TGF‐β: transforming growth factor beta
TNF‐α: tumor necrosis factor alpha
VLDL: very low density lipoprotein
α‐SMA: alpha‐smooth muscle actin

## 1 Introduction

Obesity is a multifactorial disease resulting from genetic, physiological, environmental, and psychological factors, characterized by excessive accumulation of energy in the form of body fat [1]. Over the past three decades, its prevalence has increased worldwide, reaching epidemic proportions, and it has been recognized by the World Health Organization (WHO) as one of the most significant global health challenges among adults. According to the 2023 World Obesity Atlas published by the World Obesity Federation (WOF), more than 2.6 billion people were overweight or obese in 2020, representing approximately 38% of the global population, with projections reaching 4 billion individuals (over 50%) by 2035 [2]. Obesity is strongly associated with the development of several comorbidities, including metabolic syndrome and chronic kidney disease (CKD) [3].

Evidence indicates that the risk of CKD increases proportionally with body mass index (BMI) [3, 4]. In the context of chronic hyperlipidemia, excessive lipid accumulation occurs not only in adipose tissue but also in the kidney, where lipotoxicity promotes the activation of pro‐inflammatory, pro‐fibrotic, and pro‐apoptotic pathways, ultimately leading to cellular injury and renal dysfunction [2, 4]. Additionally, the increased metabolic demand associated with elevated body mass contributes to tubular injury, glomerular hyperfiltration, and increased intraglomerular pressure, thereby enhancing the risk of kidney damage [5]. Lipid accumulation has been observed in mesangial cells, podocytes, and proximal tubular epithelial cells, the latter being particularly susceptible due to their reliance on fatty acid β‐oxidation as a primary energy source [6].

Inflammation and fibrosis represent the final common pathways of renal injury. In this context, therapeutic strategies targeting these processes, such as supplementation with omega‐3 polyunsaturated fatty acids (PUFAs), have gained considerable attention. Experimental studies using different models have demonstrated the beneficial effects of omega‐3 PUFAs in modulating inflammatory responses and improving metabolic parameters [7].

PUFAs are essential nutrients that serve as precursors for the synthesis of lipid mediators, including resolvins and lipoxins, which exhibit potent anti‐inflammatory properties. These mediators act by inhibiting the production of pro‐inflammatory cytokines, modulating transcription factor activity, regulating macrophage recruitment, and controlling the generation of reactive oxygen species and nitric oxide [8]. In addition, dietary strategies enriched in n‐3 PUFAs have been associated with improvements in hypertension and kidney disease outcomes [9]. Currently, fish oil represents the most commonly used source of PUFA supplementation [10]. However, conventional plant‐ and animal‐derived sources present several limitations, including variability in fatty acid composition, susceptibility to oxidative degradation, relatively low extraction yields, and reduced oral bioavailability of lipophilic compounds [11]. Therefore, the identification of alternative and more sustainable sources of PUFAs is of great relevance. Among the alternative animal‐derived sources currently under investigation, capybara oil has attracted increasing interest because of its lipid composition and biological properties.

Capybara oil has emerged as a promising alternative source of bioactive lipids due to its high content of unsaturated fatty acids, particularly α‐linolenic acid, which may contribute to its reported anti‐inflammatory and antioxidant effects. Previous investigations have demonstrated that this oil reduces total cholesterol and circulating levels of VLDL and LDL in rats fed a high‐cholesterol diet [12]. Furthermore, studies from our group have shown that capybara oil improves hepatic steatosis, inflammation, mitochondrial activity, and apoptosis [13, 14], and attenuates renal inflammation and pathophysiological alterations in obese mice [15]. Notably, capybara oil contains approximately 17.9% alpha‐linolenic acids (ALA), corresponding to essential n‐3 PUFAs [12].

Despite their beneficial biological properties, oils rich in PUFAs exhibit significant pharmaceutical limitations, including poor aqueous dispersibility, susceptibility to oxidation, variable gastrointestinal absorption, and, consequently, reduced bioavailability. These limitations may compromise their therapeutic efficacy after oral administration. In this context, pharmaceutical nanotechnology has emerged as an effective strategy to enhance the stability and delivery of these compounds [16]. Nanosystems exhibit specific physicochemical properties that enable a wide range of applications, including drug delivery in biomedical research. Among these systems, organic nanocarriers—such as polymeric nanoparticles and lipid‐based systems (e.g., liposomes, solid lipid nanoparticles, and nanoemulsions)—have been widely investigated. Unlike conventional oil formulations, which are often limited by poor dispersibility and oxidative instability, nanoemulsions provide a kinetically stable system capable of enhancing the delivery of lipophilic bioactive compounds. It can improve the dispersion of lipophilic compounds in aqueous media, increase the interfacial surface area, facilitating the dispersion of lipophilic compounds in aqueous media and potentially improving their gastrointestinal absorption and bioavailability. These characteristics make nanoemulsions an attractive strategy for improving the therapeutic performance of natural oils [17].

Although previous studies have demonstrated the beneficial effects of free capybara oil on experimental obesity‐associated disorders [13, 14, 15], the therapeutic potential of its nanoemulsion formulation for obesity‐induced kidney injury remains poorly understood. Therefore, in the present study, we employed a nanoemulsion formulation containing capybara oil to evaluate its impact on systemic and renal pathophysiological alterations induced by obesity in C57BL/6 mice.

## 2 Materials and Methods

### 2.1 Obtention and Characterization of Capybara Oil and Development of Nanoemulsion

Subcutaneous capybara fat was obtained from Santa Luzia Farm (Goias State, Brazil). The animals were bred in captivity under authorization from the Brazilian Institute of Environment and Renewable Natural Resources (IBAMA). The fat was extracted using hydrothermal processing in a water bath to liquid oil for oral treatment of mice with free capybara oil [13]. Part of the extracted oil was separated to produce nanoemulsions for the treatment with capybara oil nanoemulsion.

Nanoemulsions containing capybara oil were prepared by the spontaneous emulsification method. The organic phase consisted of sorbitan monostearate (40 mg) and capybara oil (components of the organic phase) (1 mg/mL), which were dissolved under magnetic stirring at 40°C in acetone (23 mL) and injected into the aqueous phase (polysorbate 80 (77 mg) and water (53 mL), under stirring and remaining in this condition for 10 min. Subsequently, the formulation was concentrated to a final volume of 10 mL in a rotary evaporator to remove the organic solvent and part of the water. The white nanoemulsion was prepared without the addition of capybara oil, which was replaced by an inert oil, medium‐chain triglyceride oil (MCT oil), called NE‐Bl [18].

The formulations were evaluated for their equivalent sphere average diameter (d4.3), calculated by Mie theory, and particle size distribution (Span) by laser diffraction (Mastersizer 2000, Malvern Instruments, United Kingdom). The measurements were performed in a wet dispersion unit containing 150 mL of water, adjusting the sample concentration to achieve an obscuration index between 2% and 8%.

The droplet size, polydispersity index (PDI), and zeta potential were analyzed using photon correlation spectroscopy following sample dilution in purified and filtered water. Zeta potential analysis was conducted based on electrophoretic mobility using a Zetasizer NanoZS model ZEN 3600 (Malvern Instruments, United Kingdom). Measurements were performed at 25°C after diluting the samples (500‐fold) in a 10 mM NaCl aqueous solution. All analyzes were carried out in triplicate across different batches to ensure reproducibility.

The pH of the nanoemulsions was determined using a potentiometer (Denver Instrument VB‐10, New York), previously calibrated with standard buffer solutions at pH 4.0, 7.0, and 9.0. Measurements were taken directly in colloidal emulsions to obtain accurate pH values.

### 2.2 Identification of Fatty Acids in Capybara Oil Using Gas Chromatography

Fatty acids were identified by gas chromatography after esterification of capybara oil according to a modified method proposed by Milinsk et al. [19]. In this process, 50 μL of the oil was combined with 0.5 mL of a 0.5 mol/L methanolic potassium hydroxide solution and subjected to heating at 70°C for 10 min. Subsequently, 400 μL of a hydrochloric acid and methanol solution (1:4 vol/vol) was incorporated into the mixture, followed by an additional heating step at 70°C for 20 min. To facilitate phase separation, 1 mL of hexane was added, and the mixture was thoroughly agitated for 5 min. After completion of the esterification process, 100 μL of the supernatant was extracted and diluted with 900 μL of hexane. The fatty acid profile of the oil was then analyzed using a gas chromatograph (Shimadzu – GCMS‐QP2010 Ultra) connected to a mass spectrometer (QP2010 Ultra), utilizing helium as the carrier gas at a flow rate of 1.0 mL min^−1^. The chromatographic conditions were set as follows: an injection volume of 1 μL, a split ratio of 3:1, an HP5‐MS column, an ion source temperature of 230°C, and a quadrupole temperature of 150°C. The temperature program consisted of an initial hold at 140°C for 2 min, a ramp of 4°C min^−1^ to 180°C, a further increase of 0.5°C min^−1^ to 200°C, and a final increase of 5°C min^−1^ to 250°C, where it was maintained for 3 min, totaling 70 min of analysis time. Fatty acid identification was performed by comparing retention times with those of standard fatty acid methyl ester (FAME) mixtures (C8:0 – C22:0, Lipid Standards Sigma‐Aldrich) analyzed under identical conditions.

### 2.3 Oil Physicochemical Characterization

The physicochemical characterization of capybara oil was performed according to AOCS methods [20]: Acidity (% of free fatty acids) as oleic acid (Ca 5a − 40), peroxide value (Cd 8b), ashes (% m/m), and moisture and melting point (capillary method) (Cs 3‐25).

#### 2.3.1 Thermogravimetric Analysis (TG) and Differential Scanning Calorimetry (DSC)

The TG curve (Shimadzu TGA‐50) was used to evaluate the oil's thermal stability, moisture, and ash content. The samples were assessed in a nitrogen atmosphere, from 30°C to 900°C, at a heating rate of 10°C min^−1^, using an alumina pan. The DSC curve was assessed with a differential scanning calorimeter (DSC 60 Plus/TAC (Shimadzu)), by weighing approximately 1 mg of the sample into a sealed aluminum pan and then performing the analysis in a nitrogen flow rate of 50 mL min^−1^ and a heating rate of 10°C min^−1^, until reaching 600°C [21].

#### 2.3.2 Fourier‐Transform Infrared Spectroscopy (FTIR)

The FTIR was used as an auxiliary method to characterize the capybara oil. The infrared spectra were obtained with a Fourier‐transform infrared spectrophotometer (Shimadzu IRAffinity‐1 (Shimadzu)) applying the sample directly to KBr tablets, at a wavelength ranging from 4000 to 500 cm^−1^, resolution of 4 cm^−1^, and 32 scans [22].

### 2.4 Experimental Groups and Diets

Forty‐eight 3‐month‐old male C57BL/6 mice were maintained under controlled temperature (21 ± 2°C) and a reversed 12 h light/dark cycle, following the Committee's guidelines. All experimental procedures involving animals were approved by the Committee for Ethics in Animal Experimentation of the University of Rio de Janeiro State (n° 048/2022). The diets were manufactured by Pragsoluções (Jau, São Paulo, Brazil) following the American Institute of Nutrition recommendations for adult rodents, AIN93M, as detailed in Table 1 [23]. The animals received 4 g of diet per day with free access to water for 18 weeks (*n* = 24 per group): control diet (CD) and high‐fat diet (HFD). The CD contained 10% of total energy from lipids, while the HFD contained 60% of total energy derived from lipids (Table 1). The HFD‐induced obesity model used in this study has been previously characterized by our group [13, 14, 15]. In the present study, successful obesity induction was confirmed by increased body mass and systolic blood pressure before treatment initiation.

**Table 1:** Composition and energy content of the experimental diets (AIN‐93M formulation).

Ingredients (g/kg)	Control diet	High‐fat diet
Casein	140	190
Corn starch	620.69	250.68
Sucrose	100	100
Lard	0	320
Soy oil	40	40
Fiber	50	50
Vitamin mix	10	10
Mineral mix	35	35
Cysteine	1.8	1.8
Choline	2.5	2.5
Antioxidants	0.008	0.016
Total mass	1000	1000
Carbohydrates (% of total energy)	76	26
Protein (% of total energy)	14	14
Lipids (% of total energy)	10	60
Total energy (kcal/kg)	3573	5404

### 2.5 Experimental Groups and Treatment Protocols

In the last 4 weeks (from weeks 15 to 18), mice were subdivided into eight groups according to treatment administration (*n* = 6 per group): CD; CD treated with free capybara oil (CD + CO); CD treated with capybara oil nanoemulsion (CD + NE‐CO); CD treated with white nanoemulsion (CD + NE‐N); HFD; HFD treated with free capybara oil (HFD + CO); HFD treated with capybara oil nanoemulsion (HFD + NE‐CO); and HFD treated with white nanoemulsion (HFD + NE‐W). Both the nanoemulsion treatment and the free capybara oil were administered once daily by oral gavage in the groups at a 100 mg/kg dose for 4 weeks. CD and HFD groups received water as a placebo to ensure similar handling among all groups.

### 2.6 Body Mass and Systolic Blood Pressure

Weekly, in the treatment period (week 15 to week 18), the body mass (BM) of the mice was measured on a precision scale to assess body weight gain.

The systolic blood pressure of the mice was measured monthly during the first 14 weeks (pre‐treatment period) by the non‐invasive method of tail‐cuff plethysmography (Kent Scientific, USA) with conscious animals. During the treatment period (from the 15th to the 18th week), SBP was measured fortnightly to assess the effect of different treatments with capybara oil on the evolution of blood pressure. The average of three measurements of each animal was used. Before the experimental period, SBP was measured in the animals to acclimatize them and minimize stress‐induced variations in blood pressure due to stress.

### 2.7 Assessment of Renal Function

One day before euthanasia, the groups of animals were placed in metabolic cages for 24‐h urine collection (*n* = 3 per cage). Urinary creatinine and protein concentrations were determined according to the manufacturer's instructions. All commercial kits used were purchased from Bioclin (Bioclin, Belo Horizonte, Minas Gerais, Brazil).

### 2.8 Euthanasia

On the day of euthanasia, 18 weeks after the administration of diets, the animals were anesthetized with an intraperitoneal injection of Ketamine and Xylazine (150 mg/kg and 15 mg/kg, respectively). Then the left kidneys were collected and frozen at −80°C for oxidative stress assays. In addition, the right kidneys were collected and fixed in 4% paraformaldehyde for light microscopy or 2.5% glutaldehyde for electron microscopy analysis.

### 2.9 Morphological Analysis

Kidneys fixed with 4% paraformaldehyde were embedded in paraffin and sectioned (3 µm thickness) as previously described in Pereira et al. [15]. The sections were stained with Hematoxylin–Eosin (H&E) for histopathological analysis, Periodic Acid‐Schiff (PAS) for glomerular basement membrane analysis, or Picrosirius Red for visualizing collagen fibrils. Then, images were observed and captured under a light microscope equipped with a CCD camera (Olympus BX53 with camera Olympus DP72, Nagano, Chubu, Japan). The Image‐Pro Plus 7.0 program (Media Cybernetics, Silver Springs, Maryland, USA) was used to obtain the HE and PAS images with a magnification of x20 and x40, respectively. Representative images were obtained under identical microscope settings from preserved areas of the renal cortex, avoiding tissue folds and processing artifacts.

### 2.10 Immunohistochemistry by Peroxidase

The sectioned kidneys (3 µm) were subjected to antigen retrieval that was performed using citrate buffer at pH 6.0, incubated for 30 min at 60°C. Endogenous peroxidase activity was blocked using 0.3% hydrogen peroxide (H_2_O_2_), and non‐specific binding of the polyclonal antibodies was blocked by incubation with 5% (wt/vol) BSA. Subsequently, sections were incubated with antibodies, and these reactions were amplified using a biotin‐streptavidin system (Dako, Santa Clara, California, USA). Immunoreactive products were visualized using diaminobenzidine (DAB) reagent (Dako) and counterstained with hematoxylin. Control sections were obtained by primary antibody omission. We used anti‐TNF‐α (sc‐52746), anti‐TGF‐β (sc‐130348), IL‐10 (sc‐8438), and anti‐α‐SMA (sc‐53142) (dilution 1:200, Santa Cruz Biotechnology, Dallas, Texas, USA) antibodies. For each animal, one histological section was analyzed, and ten non‐overlapping random fields were randomly selected from the renal cortex while avoiding tissue folds, large blood vessels, and section artifacts. Images were acquired under identical illumination and microscope settings for all experimental groups using magnifications of ×20 or ×40, according to the histological marker analyzed. Semi‐quantitative analysis was performed using Image‐Pro Plus 7.0 software (Media Cybernetics, Silver Spring, MD, USA). Immunoreactivity was quantified by determining the absolute number of positively stained pixels within the analyzed field after color threshold segmentation. The same threshold parameters were applied to all images for each immunomarker to ensure consistency among the experimental groups. Image acquisition and analysis were performed in a blinded manner.

### 2.11 Oxidative Stress Assay

Kidney homogenates were used for biochemical assays, as performed by Kennedy‐Feitosa et al. [24]. Superoxide dismutase (SOD) activity was assayed by monitoring adrenaline (Sigma‐Aldrich, USA) inhibition of auto‐oxidation. Catalase (CAT) activity was measured by a decrease in H_2_O_2_ (Sigma‐Aldrich, USA) concentration, and concentrations were monitored.

### 2.12 Electron Microscopic Analysis

Kidneys were collected, cut into small tissue blocks (1 mm^3^), and fixed in 2.5% glutaraldehyde at 4°C. For transmission electron microscopy (TEM), after postfixation with 2% osmium tetroxide, tissues were dehydrated in a graded series of acetone and embedded in epoxy resin, as performed by Lontro Alves et al. [14]. Ultrathin sections were double stained with uranyl acetate and lead citrate and examined using a JEM1011 transmission electron microscope (JEOL, Peabody, MA, USA) operating at 80 kV. Three animals from each experimental group were processed for ultrastructural analysis. Representative images were acquired from preserved regions of the renal cortex, including glomeruli and proximal tubules, while avoiding areas containing processing artifacts. Ultrastructural evaluation was performed qualitatively to assess renal cellular and subcellular morphology.

For scanning electron microscopy (SEM), the fragments were washed in 0.1 M sodium cacodylate buffer and postfixed with 1% osmium tetroxide diluted in 0.1 M sodium cacodylate buffer. After further washing, the material was dehydrated in an increasing series of ethanol (30%, 50%, 70%, 90%, and 2x absolute), 30 min each step. The material was taken to the critical point device for the replacement of ethanol by CO_2_, mounted on aluminum stubs with carbon tape, and sputter‐coated with gold. After metallization, the material was analyzed using a scanning electron microscope JEOL‐JSM‐6390‐LV (JEOL, Peabody, USA), at 25 kV. Similarly, three animals from each experimental group were processed for scanning electron microscopy. Representative images were obtained from preserved cortical regions free of preparation artifacts. The analysis was performed qualitatively to evaluate renal surface ultrastructural features.

### 2.13 Western Blot Analysis

RIPA‐extracted renal cortical proteins were subjected to SDS‐PAGE and transferred to nitrocellulose membranes. After blocking with 5% BSA in TBS‐T for 1 h, membranes were probed overnight with primary antibodies (1:1000) against p‐NF‐κB p65 (Ser536) (sc‐136548) and β‐actin (sc‐47778). Following incubation with an HRP‐conjugated secondary antibody (1:10,000; BA:1050), immunoreactive bands were detected by enhanced chemiluminescence using a ChemiDoc MP Imaging System (Bio‐Rad, USA). Densitometric analysis was performed using ImageJ (NIH, USA), and bands expression was normalized to β‐actin.

### 2.14 Statistical Analysis

Data are presented as mean ± standard deviation. Statistical analyzes were performed by comparing the groups, and differences between them were tested. Data were compared by Student's t‐test (pre‐treatment period) or two‐way ANOVA (treatment period) followed by Holm‐Sidak's post‐test. The significance level used was 5%. GraphPad Prism version 8.0 (GraphPad Software, San Diego, CA, USA) was used to perform statistical analysis and graphing.

## 3 Results

### 3.1 Physicochemical Characterization of Capybara Oil

The organoleptic analysis of capybara oil showed that, at 25°C, it was liquid, clear, with a citrine yellowish color, consistent with the fatty acid profile described below (Table 2).

**Table 2:** Physicochemical parameters of capybara oil. The calculated deviation is the relative standard deviation.

Parameters	Values
Acidity (% oleic acid)	0.378 ± 1.68
Peroxide index (meq/kg)	5.08 ± 0.712
Ashes (% m/m)^a^	6.84
Moisture^a^	Not detected
Melting point^b^	38°C

^a^ Determined by TG curve.

^b^ determined by DSC curve.

The physicochemical analysis revealed an acidity index of 0.378 ± 1.68, consistent with the oleic acid content (3.76%) described in Table 2 and within the reference limits established by Normative Instruction (NI) No. 49/2006. The peroxide index (5.08 ± 0.712) indicated low rancidity, remaining below the maximum limit of 20 meq/kg defined by the same regulation. The absence of moisture (Figure 1A) supports these findings, as water content can promote lipid hydrolysis and oxidation of polyunsaturated fatty acids. The total ash content was 6.84%, indicating the presence of inorganic residues and/or impurities. This relatively high value may be associated with the non‐refined nature of the oil and the lack of standardization in the extraction process (Table 2).

**Figure 1:**
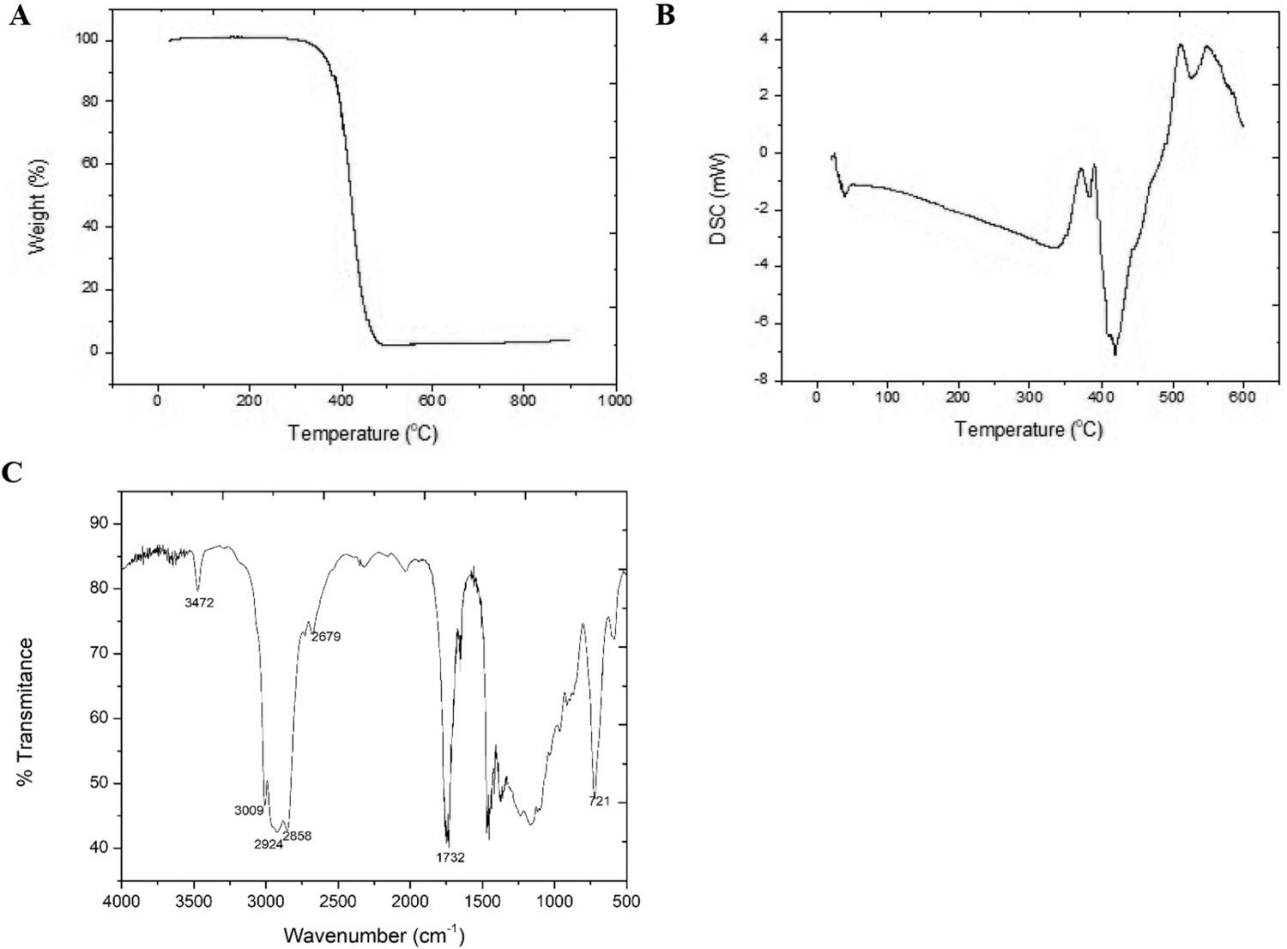
Characterization of capybara oil (A) Thermogravimetric (TG) curve of Capybara oil. (B) Differential scanning calorimetry (DSC) curve of the Capybara oil. (C) Fourier‐transform infrared spectrum of the Capybara oil.

Thermogravimetric analysis (TG) (Figure 1A) confirmed the absence of moisture and demonstrated that the oil remained thermally stable up to 295°C. Above this temperature, a mass loss of 93.16% was observed, corresponding to decomposition and/or oxidation processes occurring in a single degradation stage up to 550°C.

Differential scanning calorimetry (DSC) (Figure 1B) showed both endothermic and exothermic events. An initial endothermic peak at 38°C corresponded to the melting point of the oil. Additional endothermic and exothermic events above 300°C were associated with degradation processes, consistent with the TG results.

FTIR analysis (Figure 1C) confirmed the lipid composition of the sample, with characteristic absorption bands at 2924 and 2858 cm^−1^ (C─H stretching), 1732 cm^−1^ (C═O stretching), and bands between 1500–1250 cm^−1^ and 1100–1000 cm^−1^ corresponding to ester functional groups. The band at 721 cm^−1^ is associated with CH_2_ rocking vibrations [25]. The absorption band at 3472 cm^−1^ suggests hydrogen bonding, possibly related to carboxylic acids or alcohol groups. The absence of moisture was further supported by the absence of mass loss in the TG curve or DSC endothermic events related to water.

### 3.2 Physicochemical Characterization of Nanoemulsion

The physicochemical characteristics of the nanoemulsions were also evaluated. The organoleptic characteristics of capybara oil and nanoemulsions containing capybara oil and inert oil (white nanoemulsion–NE‐W) were analyzed, which served as the vehicle control (Table 3).

**Table 3:** Physicochemical characterization of white nanoemulsion (NE‐W) and nanoemulsion containing capybara oil (NE‐CO).

	Characteristics	Results	
		NE‐W	NE‐CO
Laser diffraction	Equivalent spherical diameter (d4.3) (µm)	0.186 ± 0.012	0.228 ± 0.041
	Particle size distribution (Span)	1.725 ± 0.025	0.651 ± 0.039
Photon correlation spectroscopy	Mean droplet diameter (Z‐average) (nm)	175.0 ± 0.007	183.9 ± 2.37
	Polydispersity index (PDI)	0.089 ± 0.021	0.109 ± 0.022
	Zeta potential (mV)	−6.72 ± 0.13	−9.43 ± 0.68
	pH	5.44 ± 0.27	5.57 ± 0.11

Abbreviations: µm, micrometer; mV: millivolt; nm, nanometer.

Both nanoemulsion formulations (NE‐W and NE‐CO) were liquid, milky‐white dispersions without visible phase separation or precipitate formation. With a milky white color without precipitate deposition. The physicochemical properties were evaluated by laser diffraction and photon correlation spectroscopy. For evaluating the physicochemical properties of nanoemulsions—laser diffraction analysis and photon correlation spectroscopy—the NE‐W nanoemulsions showed an equivalent spherical diameter of 0.186 ± 0.012 μm and 175.0 ± 0.007 nm, respectively. The analysis of nanoemulsions containing capybara oil (NE‐CO) showed an equivalent spherical diameter of 0.228 ± 0.041 μm and 183.9 ± 2.37 nm, respectively, between the particles. Particle homogeneity was further evaluated by homogeneity and distribution amplitude of the particles in the formulations, which were evaluated through parameters such as the particle size distribution (Span) and the polydispersity index (PDI). The NE‐W formulation presented a Span value of 1.725 ± 0.025 and a PDI of 0.089 ± 0.021. The NE‐CO nanoemulsion presented a particle size distribution of 0.651 ± 0.039 Span and PI 0.109 ± 0.022. Both formulations exhibited slightly acidic pH values (NE‐W: 5.44 ± 0.27 and NE‐CO: 5.57 ± 0.11) and negative zeta potential values close to neutrality (NE‐W: −6.72 ± 0.13 and NE‐CO: −9.43 ± 0.68). The low PDI values indicate good homogeneity of the dispersed systems, while the nanometric droplet size is consistent with stable nanoemulsion formulations described in the literature [17].

### 3.3 Anthropometric Measurement and Systolic Blood Pressure

Body mass (BM) and systolic blood pressure (SBP) were monitored throughout the experimental period. As previously reported by our group, during the pre‐treatment phase, BM remained stable in the CD group, whereas HFD‐fed animals showed a significant increase compared with controls [15]. During the treatment period (weeks 15–18; Figure 2A), BM remained stable in the CD‐fed groups, whereas HFD‐fed mice maintained significantly higher values. Notably, the HFD + NE‐CO group showed a significant reduction in BM at weeks 17 and 18 compared with the HFD group.

**Figure 2:**
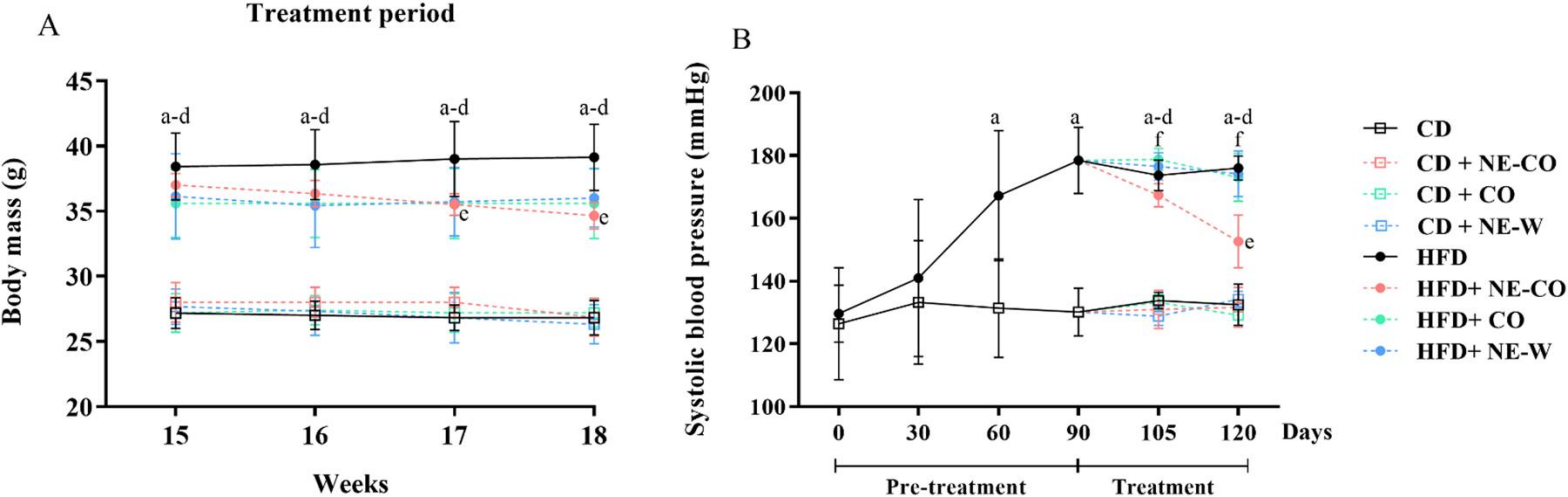
Evolution of body mass and systolic blood pressure. (A) Body mass during the treatment period in grams (g). (B) Systolic blood pressure measured in millimeters of mercury (mmHg) in the pre‐treatment and treatment periods. (a) *p* < 0.05 versus CD group, (b) *p* < 0.05 versus CD + NE‐CO group, (c) *p* < 0.05 versus CD + CO group, (d) *p* < 0.05 versus CD + NE‐W group, (e) *p* < 0.05 versus HFD group and (f) *p* < 0.05 versus HFD + CO group. *n* = 6 for all experimental groups.

SBP was measured monthly during the pre‐treatment period (weeks 0–14) and biweekly during treatment (Figure 2B). During the pre‐treatment phase, SBP progressively increased in HFD‐fed animals while remaining stable in the CD group. During treatment, no significant differences in SBP were observed among the CD‐fed groups (CD, CD + NE‐CO, CD + CO, and CD + NE‐W), whereas the HFD‐fed groups maintained significantly higher SBP values compared with the control diet groups. However, the HFD + NE‐CO group showed a significant reduction in SBP in the final week of treatment compared with the untreated HFD group.

### 3.4 Assessment of Renal Function

Urinary protein and creatinine levels were evaluated across the experimental groups (Figure 3). Protein excretion was significantly increased in the HFD, HFD + CO, and HFD + NE‐W groups compared with the control diet groups. In contrast, the HFD + NE‐CO group showed significantly reduced protein excretion, with values comparable to those observed in the control diet groups (Figure 3A). Similarly, urinary creatinine levels were significantly reduced in the HFD, HFD + CO, and HFD + NE‐W groups compared with the control diet groups. However, the HFD + NE‐CO group showed significantly increased urinary creatinine levels compared with the other HFD‐fed groups, reaching values comparable to those observed in the control diet groups (Figure 3B).

**Figure 3:**
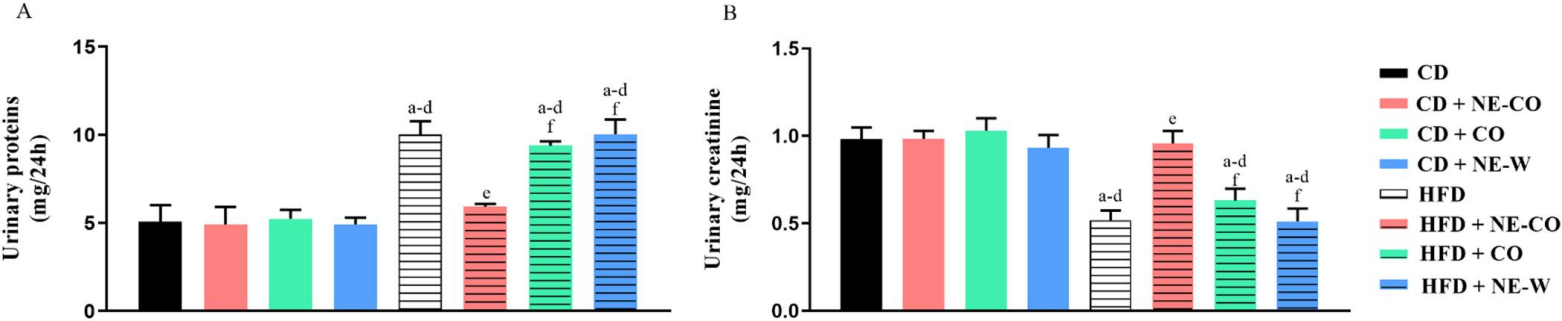
Urinary biochemical parameters. (A) Protein excretion (mg/24 h) and (B) Urinary creatinine (mg/dL). (a) *p* < 0.05 versus CD group, (b) *p* < 0.05 versus CD + NE‐CO group, (c) *p* < 0.05 versus CD + CO group, (d) *p* < 0.05 versus CD + NE‐W group, (e) *p* < 0.05 versus HFD group and (f) *p* < 0.05 versus HFD + CO group. *n* = 6 for all experimental groups.

### 3.5 Histopathological Evaluation

Hematoxylin and eosin (H&E) staining was used to evaluate renal histopathology (Figure 4A). The control diet groups exhibited well‐preserved renal corpuscles and proximal and distal convoluted tubules. In contrast, the HFD, HFD + CO, and HFD + NE‐W groups showed loss of renal corpuscle and tubular organization, as well as areas suggestive of lipid vesicles. The HFD + NE‐CO group exhibited fewer areas suggestive of lipid vesicles and improved preservation of the renal cortex, particularly the renal corpuscles and proximal and distal convoluted tubules, compared with the other HFD‐fed groups.

**Figure 4:**
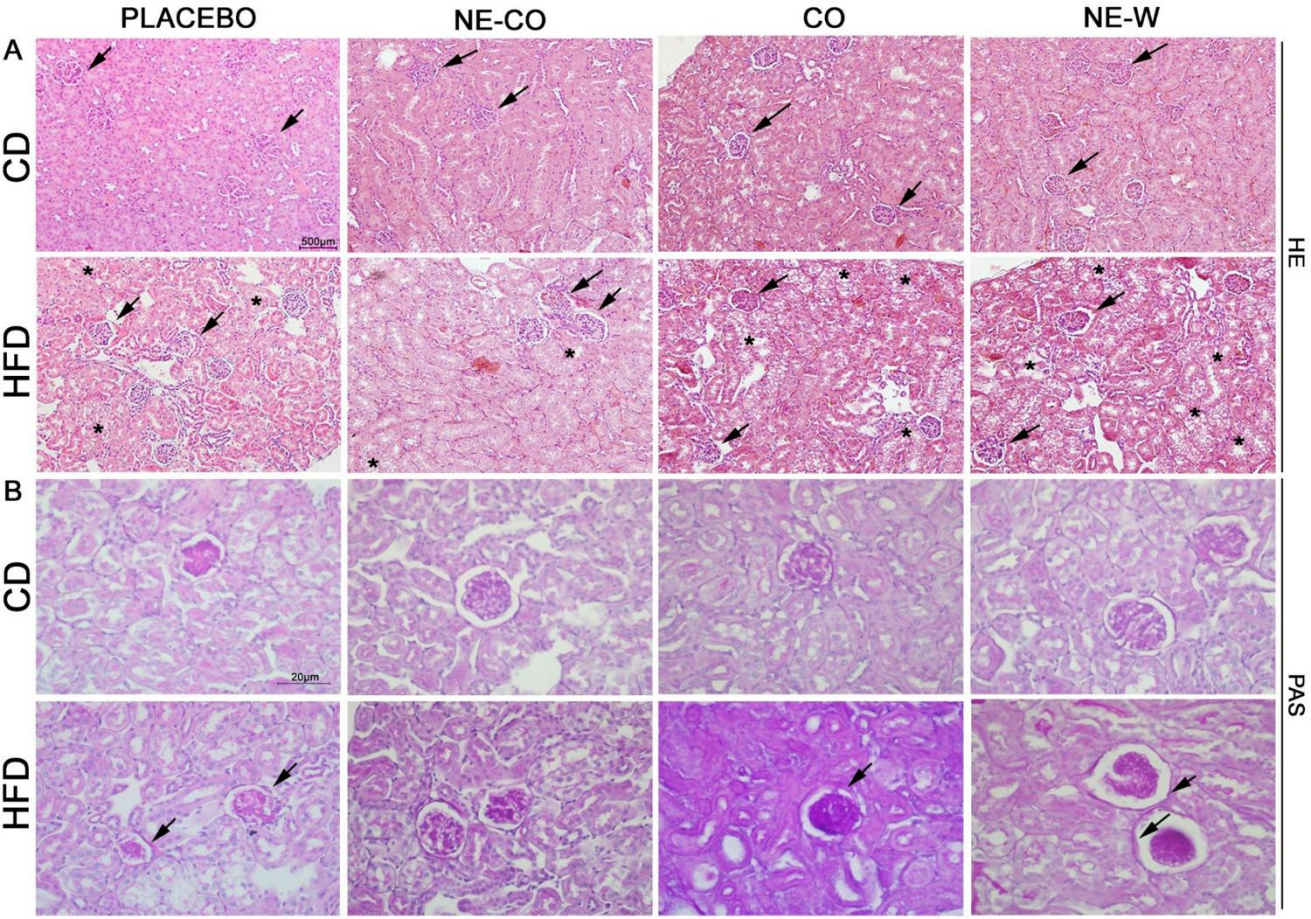
Renal histopathology. (A) Representative photomicrographs of hematoxylin and eosin (H&E) staining. (B) Representative photomicrographs of periodic acid–Schiff (PAS) staining. In H&E‐stained sections, arrows indicate glomeruli and asterisks indicate possible areas containing lipid vesicles. In PAS‐stained sections, arrows indicate glomerular basement membrane thickening. Scale bars: 500 µm (A) and 20 µm (B).

PAS staining was performed to evaluate the basement membranes of the glomerular capillary loops and tubular epithelium (Figure 4B). The HFD, HFD + CO, and HFD + NE‐W groups exhibited more intense PAS staining in the basement membranes, particularly in the parietal layer of Bowman's capsule and glomerular capillaries, compared with the control diet groups. In contrast, the HFD + NE‐CO group showed preservation of the basement membranes of Bowman's capsule, glomeruli, and proximal and distal convoluted tubules, with staining comparable to that observed in the control diet groups.

### 3.6 Evaluation of Renal Ultrastructure

Scanning electron microscopy revealed that the renal ultrastructure of the control groups presented intact podocytes (asterisk) comprising primary and secondary processes (pedicels) (arrow), in addition to the cell body (star), and the filtration slits were preserved between the regular interdigitations of the pedicels. The HFD, HFD + CO, and HFD + NE‐W groups exhibited more dilated filtration slits (arrowhead) and pedicels (arrow) with irregular contours, in addition to many pedicels lacking regular interdigitations, as well as thickening of the primary extensions (asterisk). In the HFD + NE‐W group, pedicels with dilated ends (arrow) were also observed, in addition to their shortening, and the HFD + CO group, thinner pedicels (arrow) with an irregular morphology, impairing the formation of regular interdigitations, in addition to shorter primary extensions (asterisk) were also observed. Treatment with NE‐CO (HFD + NE‐CO) improved ultrastructural preservation of the primary (asterisk) and secondary (arrow) extensions, with regular interdigitations of the pedicels, with thinner and more elongated primary processes compared to the other groups that received a high‐fat diet (Figure 5).

**Figure 5:**
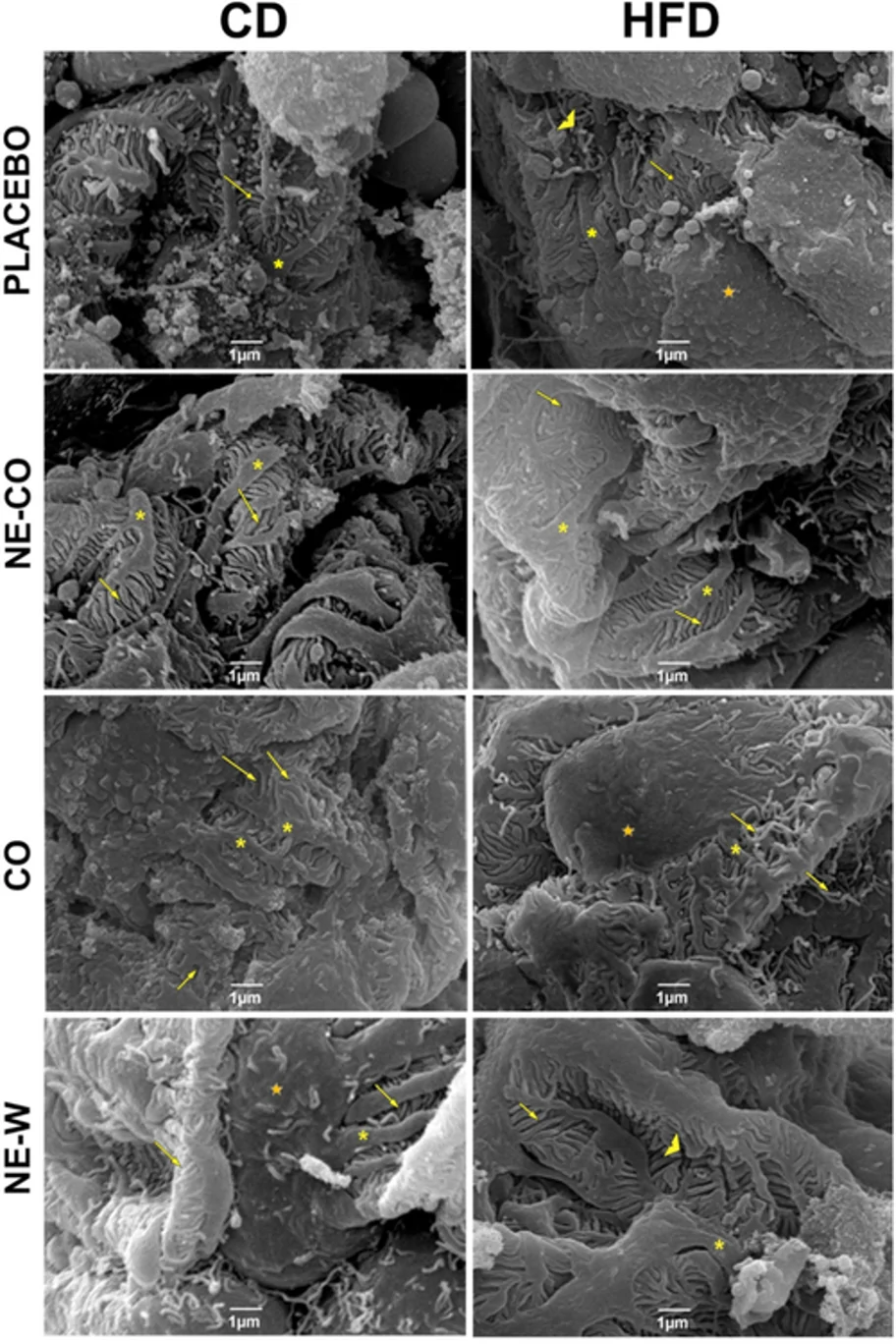
Renal ultrastructure evaluated by scanning electron microscopy. Representative electron micrographs of the glomerular filtration barrier in the experimental groups. Podocytes (*), pedicels (arrows), cell body (star), and glomerular filtration cleft dilatation (arrowhead) are indicated. Scale bar: 1 µm.

Transmission electron microscopy demonstrated that (Figure 6) animals in groups CD, CD + NE‐CO, CD + CO, and CD + NE‐W presented numerous pedicels (arrowhead) with uniform thickness forming filtration slits (arrow) with evident membranes. In these groups, the glomerular basement membrane (asterisk) and the pores of the glomerular capillaries (cross) were intact. In the groups HFD, HFD + CO, and HFD + NE‐W, ultrastructural alterations were observed such as thickening of the glomerular basement membrane (asterisk), variable and irregular size of the pedicels (arrowhead), in which pedicels with different thicknesses are observed. Additionally, discontinuity of the fenestrated glomerular capillary (cross) pores was observed. Furthermore, in the HFD + CO group, the glomerular pore membrane was absent, as well as the absence of the glomerular filtration slit membrane, whereas in the HFD + NE‐W group, absence of fenestrated glomerular capillary pores and shorter pedicels. The HFD + NE‐CO showed improved ultrastructural preservation with the remodeling of the pedicels (arrowhead), filtration slits (arrow), and preserved membranes. Furthermore, the integrity of the glomerular basement membrane (asterisk) was observed, as well as the continuity of the glomerular capillary pores (cross) compared with the groups that ingested the high‐fat diet.

**Figure 6:**
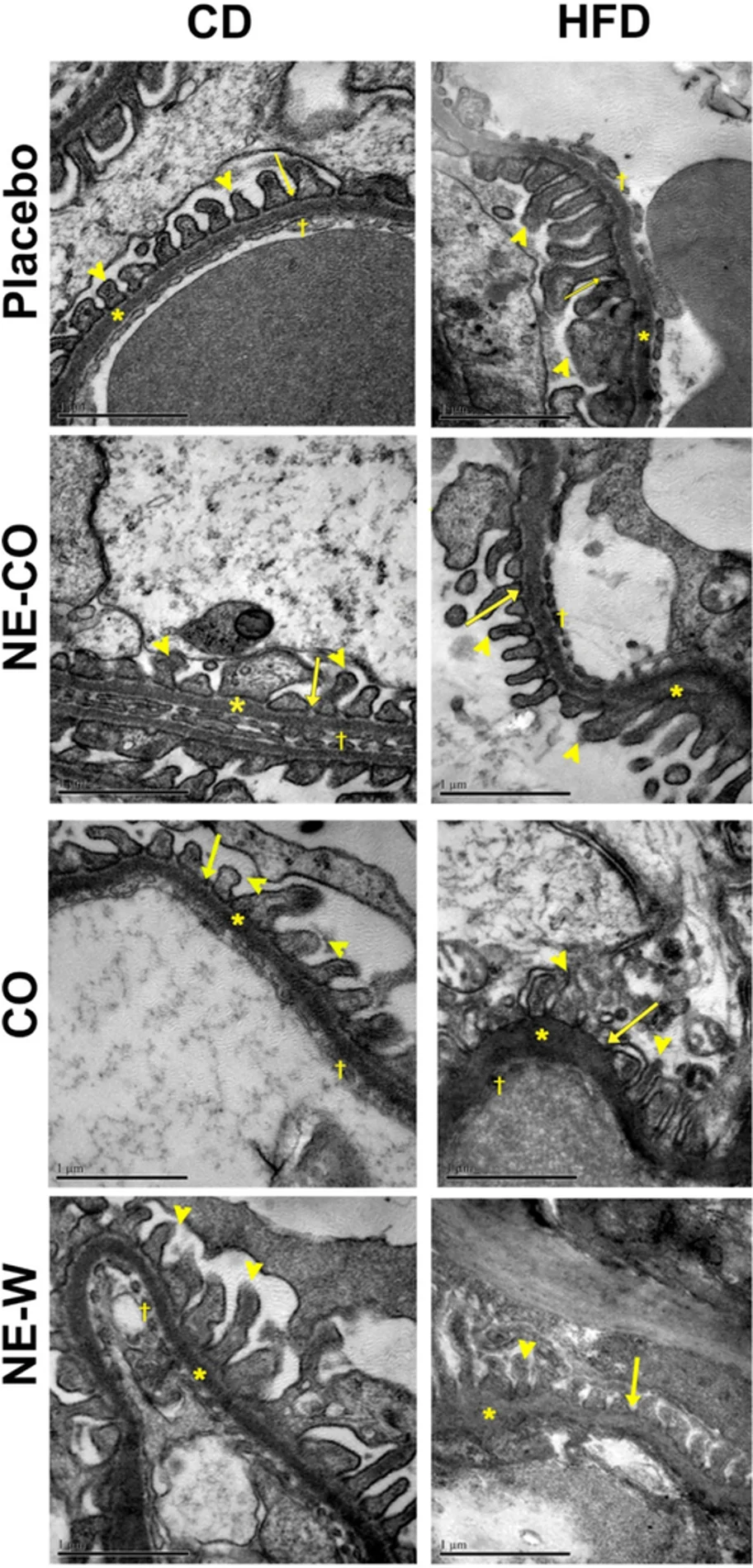
Renal ultrastructure evaluated by transmission electron microscopy. Representative electron micrographs of the glomerular filtration barrier in the experimental groups. Arrows indicate filtration slits, arrowheads indicate pedicels, (*) indicates the glomerular basement membrane, and (†) indicates pores of the fenestrated glomerular capillaries. Scale bar: 1 µm.

### 3.7 Renal Inflammatory Response and p‐NF‐κB Expression

Immunostaining for TNF‐α and IL‐10 revealed changes in the renal inflammatory response among the experimental groups (Figure 7). TNF‐α immunostaining was increased in the renal cortex of the HFD, HFD + CO, and HFD + NE‐W groups, particularly in the glomeruli and proximal and distal tubules, whereas the HFD + NE‐CO group showed reduced TNF‐α immunostaining (Figure 7A, C). Conversely, IL‐10 immunostaining was increased in the HFD + NE‐CO group compared with the other HFD‐fed groups (Figure 7B, D). No significant differences in TNF‐α or IL‐10 immunostaining were observed among the control diet groups.

**Figure 7:**
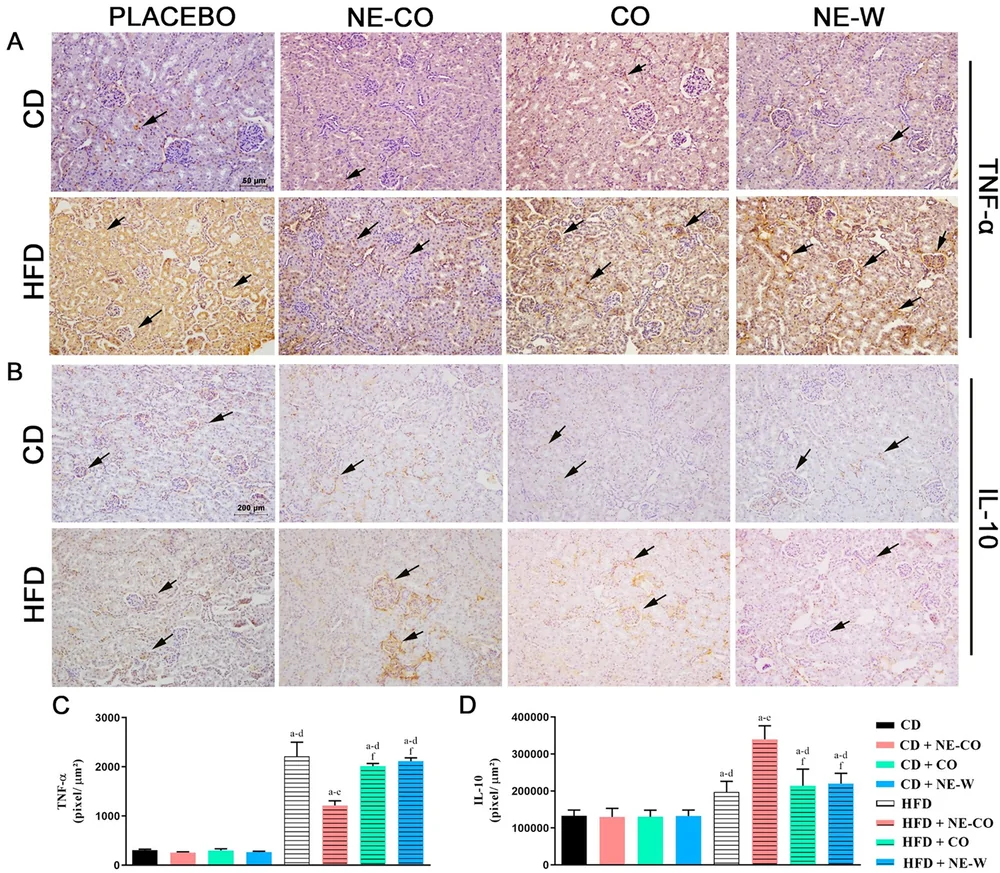
Immunostaining and quantification of TNF‐α and IL‐10 cytokines in the renal cortex of the experimental groups. (A) Representative photomicrographs of TNF‐α immunostaining. (B) Representative photomicrographs of IL‐10 immunostaining. Arrows indicate positive immunostaining. (C) Represents quantification of TNF‐α‐positive immunostaining and (D) Represents quantification of IL‐10‐positive immunostaining, expressed as pixels/µm^2^. Scale bars: 50 µm (A) and 200 µm (B). Data are expressed as mean ± SEM (*n* = 6 per group). (a) *p* < 0.05 versus CD; (b) *p* < 0.05 versus CD + NE‐CO; (c) *p* < 0.05 versus CD + CO; (d) *p* < 0.05 versus CD + NE‐W; (e) *p* < 0.05 versus HFD; and (f) *p* < 0.05 versus HFD + CO.

Western blot analysis revealed significant differences in renal p‐NF‐κB expression among the experimental groups (Figure 8). Renal p‐NF‐κB expression was increased in the HFD, HFD + CO, and HFD + NE‐W groups compared with the CD group. In contrast, the HFD + NE‐CO group showed significantly reduced p‐NF‐κB expression compared with the HFD, HFD + CO, and HFD + NE‐W groups.

**Figure 8:**
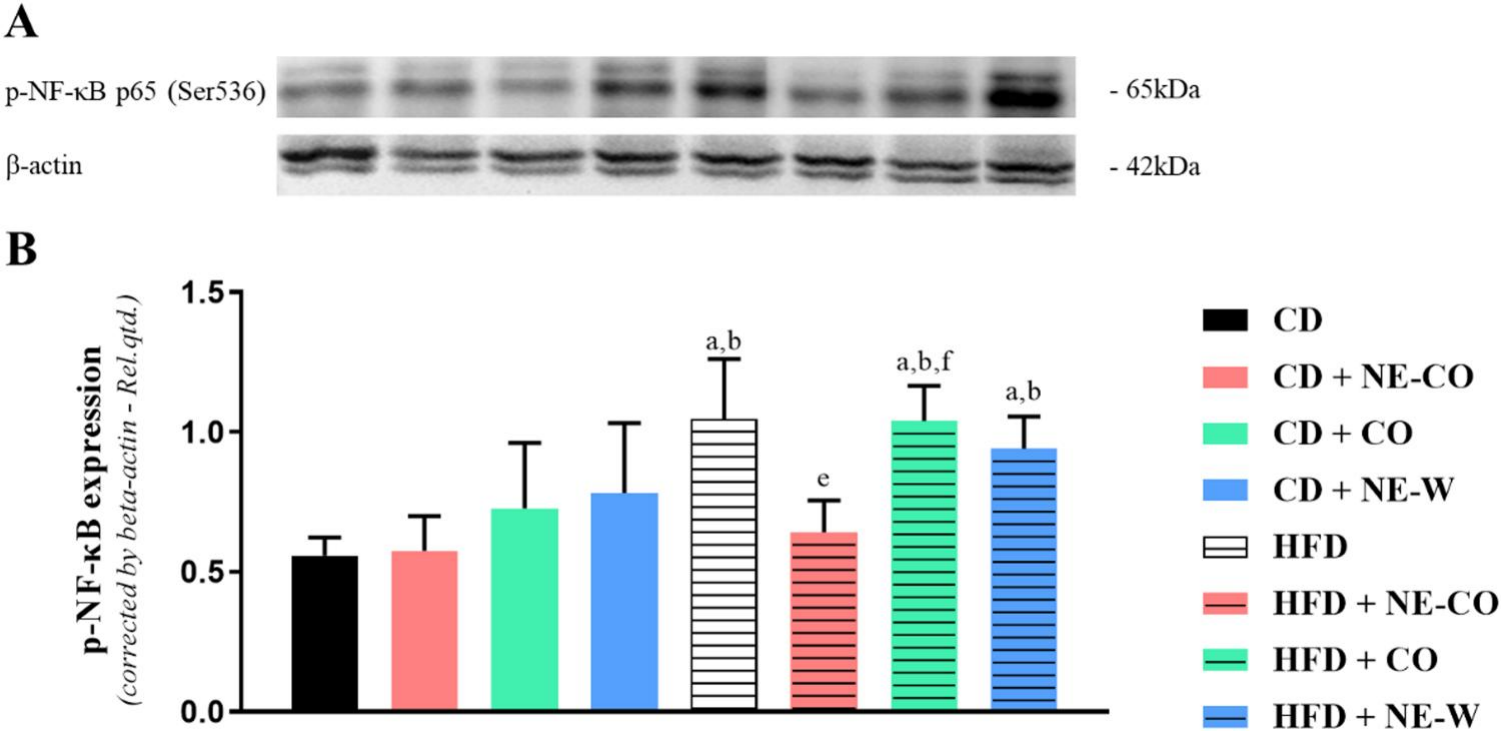
Renal p‐NF‐κB p65 (Ser536) expression in the experimental groups. (A) Representative Western blot bands of p‐NF‐κB p65 (Ser536) and β‐actin. (B) Densitometric analysis of p‐NF‐κB p65 (Ser536) expression normalized to β‐actin. Data are expressed as mean ± SEM (*n* = 6 per group). (a) *p* < 0.05 versus CD; (b) *p* < 0.05 versus CD + NE‐CO; (c) *p* < 0.05 versus CD + CO; (d) *p* < 0.05 versus CD + NE‐W; (e) *p* < 0.05 versus HFD; and (f) *p* < 0.05 versus HFD + CO.

### 3.8 Analysis of the Activity of Enzymes Related to Oxidative Stress

Analysis of antioxidant enzyme activity revealed significant differences among the experimental groups (Figure 9). No significant differences in CAT or SOD activity were observed among the control diet groups (CD, CD + NE‐CO, CD + CO, and CD + NE‐W). In contrast, the HFD, HFD + CO, and HFD + NE‐W groups showed significantly increased CAT (Figure 9A) and SOD (Figure 9B) activity. The HFD + NE‐CO group exhibited significantly lower CAT and SOD activity than the other HFD‐fed groups.

**Figure 9:**
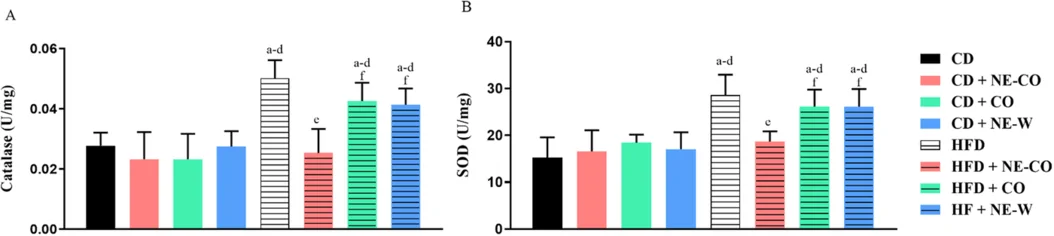
Antioxidant enzyme activity (U/mg). (A) Catalase (CAT) activity and (B) Superoxide dismutase (SOD) activity. (a) *p* < 0.05 versus CD group, (b) *p* < 0.05 versus CD + NE‐CO group, (c) *p* < 0.05 versus CD + CO group, (d) *p* < 0.05 versus CD + NE‐W group, (e) *p* < 0.05 versus HFD group and (f) *p* < 0.05 versus HFD + CO group. *n* = 6 for all experimental groups.

### 3.9 Renal Fibrosis Analysis

Collagen fiber deposition was evaluated by Picrosirius Red (PSR) staining (Figure 10A). Increased collagen deposition was observed in the HFD, HFD + CO, and HFD + NE‐W groups, whereas the HFD + NE‐CO group showed only mild collagen deposition. Immunohistochemical analysis revealed increased TGF‐β and α‐SMA immunostaining in the HFD‐fed groups compared with the control diet groups (Figure 10B, C). However, the HFD + NE‐CO group exhibited reduced TGF‐β and α‐SMA immunostaining compared with the other HFD‐fed groups. Quantitative analysis confirmed these findings, showing significantly increased TGF‐β and α‐SMA immunostaining in the HFD, HFD + CO, and HFD + NE‐W groups (Figure 10D,E). In contrast, the HFD + NE‐CO group showed significantly lower immunostaining for both markers compared with the HFD group.

**Figure 10:**
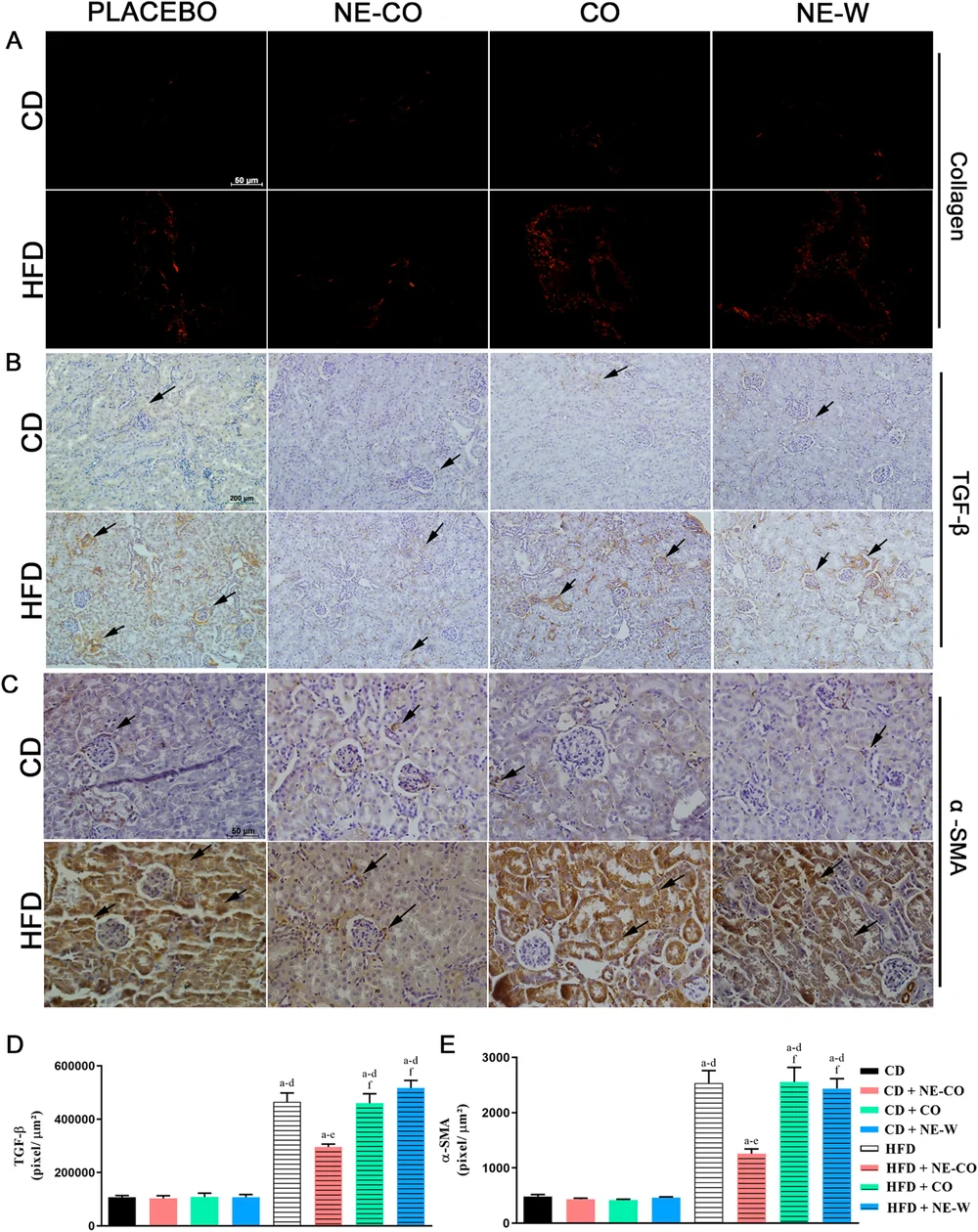
Renal fibrosis analysis in all groups. (A) Representative photomicrographs of Picrosirius Red (PSR) staining for collagen fibers. (B) Representative photomicrographs of TGF‐β immunostaining. (C) Representative photomicrographs of α‐SMA immunostaining. Arrows indicate positive immunostaining. (D) Quantification of TGF‐β‐positive immunostaining and (E) quantification of α‐SMA‐positive immunostaining, expressed as pixels/µm^2^. Scale bars: 50 µm (A and C) and 200 µm (B). Data are expressed as mean ± SEM (*n* = 6 per group). (a) *p* < 0.05 versus CD; (b) *p* < 0.05 versus CD + NE‐CO; (c) *p* < 0.05 versus CD + CO; (d) *p* < 0.05 versus CD + NE‐W; (e) *p* < 0.05 versus HFD; and (f) *p* < 0.05 versus HFD + CO.

## 4 Discussion

Capybara oil exhibited thermal and spectroscopic characteristics consistent with triglyceride‐rich oils. FTIR confirmed typical lipid functional groups, while TGA and DSC analyzes demonstrated thermal stability, with degradation occurring between 300°C and 500°C and defined melting and oxidation events. These properties support its suitability for pharmaceutical applications, particularly in nanoemulsion systems.

Nanoemulsions are widely used drug delivery systems due to their favorable physicochemical properties [16]. In the present study, both formulations exhibited nanometric size distribution, with most droplets around 70 nm, classifying them as nanomaterials according to European Union criteria. The negative zeta potential and stable pH values indicate good colloidal stability [25]. Additionally, their monodispersity and reduced particle size may enhance bioavailability, allowing lower doses and improved therapeutic efficiency [26].

Consistent with previous studies, prolonged high‐fat diet (HFD) intake induced obesity and systemic alterations, including increased body mass (BM), systolic blood pressure (SBP), and renal dysfunction [27]. These effects are associated with a chronic pro‐inflammatory and pro‐fibrotic state, leading to structural and functional kidney alterations characteristic of obesity‐associated glomerulopathy [28]. PUFA‐rich oils have shown beneficial effects in obesity models [10, 29], likely through modulation of signaling pathways and production of bioactive lipid mediators [29]. In the kidney, fatty acid‐derived eicosanoids regulate glomerular hemodynamics and fluid balance via the COX pathway [4], supporting the relevance of lipid‐based interventions in renal disease [30]. Given the limitations associated with marine‐derived n‐3 sources [10], capybara oil represents a promising alternative. Previous studies have reported beneficial effects of this capybara by‐product, including improvements in steatosis, inflammation, mitochondrial activity, and apoptosis in the liver of C57BL/6 mice [13, 14], as well as renal pathophysiology and inflammation in obese mice [15].

In agreement with the literature, HFD‐fed mice exhibited increased BM and SBP [25, 31], reinforcing the well‐established association between obesity and hypertension. Epidemiological data indicate that obese individuals are approximately 3.5 times more likely to develop hypertension, with nearly 60% of cases attributable to excess adiposity [32]. This relationship is complex and multifactorial, involving sympathetic nervous system activation, endothelial dysfunction, and metabolic disturbances. In this context, dietary PUFA intake has been associated with improved blood pressure regulation [33], supporting the effects observed in the present study.

Lipotoxicity plays a central role in obesity‐induced renal injury, promoting inflammation, oxidative stress, fibrosis, and apoptosis [34]. Accordingly, we observed structural and ultrastructural renal alterations, together with impaired renal function, as indicated by reduced creatinine excretion and increased proteinuria [35]. Both alterations were reversed by NE‐CO treatment, whereas free capybara oil did not significantly affect these parameters in previous work [15], highlighting the improved efficacy of the nanoemulsion. Morphological analysis further revealed glomerular damage in HFD animals, including basal lamina thickening and podocyte alterations that compromise the filtration barrier [36]. NE‐CO treatment restored these structures, indicating improved glomerular integrity.

Oxidative stress, driven by excessive ROS production, is a key mechanism in obesity‐related damage [37]. Increased SOD and CAT activity in obese animals may reflect an adaptive alteration in the antioxidant response [38, 39]. However, without direct measurements of oxidative products, these findings should not be interpreted as direct evidence of oxidative damage. NE‐CO treatment normalized these enzymatic responses and reduced TNF‐α expression while increasing IL‐10 expression, indicating modulation of the inflammatory response. Renal p‐NF‐κB expression was also increased in HFD‐fed animals and significantly reduced following NE‐CO treatment, providing additional molecular support for the observed modulation of inflammation. TNF‐α is a major mediator of renal injury, whereas IL‐10 exerts protective anti‐inflammatory effects [40]. The interaction between oxidative stress and inflammation contributes to the progression of renal damage [41, 42]. Fibrosis represents an important downstream consequence, with TGF‐β and α‐SMA acting as key mediators of extracellular matrix deposition and myofibroblast activation [43, 44, 45], processes closely associated with epithelial–mesenchymal transition (EMT) [46, 47]. Consistently, NE‐CO reduced TGF‐β and α‐SMA expression, indicating attenuation of fibrotic responses.

The beneficial effects of NE‐CO may also be related to the lipid composition previously described for capybara oil. Fukushima et al. [12] reported its n‐3 and n‐6 fatty acid composition; however, these components could not be quantified in the present study because the analytical standards required to generate a reference curve were unavailable. Saturated fatty acids, such as palmitic, stearic, and myristic acids, can contribute to the pro‐inflammatory state observed in obesity, partly through NF‐κB activation and subsequent production of pro‐inflammatory cytokines [48]. In contrast, PUFA‐enriched diets, particularly those containing EPA and DHA, exert anti‐inflammatory effects by inhibiting n‐6‐derived eicosanoids and reducing cytokine production. Additionally, n‐3 PUFA compete with n‐6 PUFA for incorporation into cell membranes via COX‐mediated pathways [49]. ALA serves as a precursor for EPA and DHA, which give rise to bioactive lipid mediators, including resolvins, protectins, maresins, and lipoxins, involved in inflammation resolution through modulation of neutrophil chemotaxis, phagocytic activity, and pro‐inflammatory cytokine production [50]. Consistently, EPA has been shown to reduce TNF‐α expression through inhibition of NF‐κB activation [51], while n‐3 PUFA administration decreases pro‐inflammatory mediators, including IL‐1, MCP‐1, MIP‐1, and TGF‐β, in renal injury models [52]. Although the specific PUFA concentrations in the capybara oil batch used in the present study were not determined, the observed anti‐inflammatory and antifibrotic effects may be consistent with biological activities previously associated with PUFA‐rich oils. However, the contribution of specific fatty acids to the effects observed here cannot be established.

This study has some limitations. A complete quantitative fatty acid profile of the capybara oil batch was not obtained because the analytical standards required for n‐3 fatty acid quantification were unavailable. Although the nanoemulsion exhibited favorable physicochemical characteristics at the time of characterization, long‐term stability and residual organic solvent content were not assessed, and dedicated in vitro or in vivo toxicological assays were not performed. Regarding molecular mechanisms, p‐NF‐κB p65 (Ser536) analysis provided additional evidence supporting modulation of the inflammatory response by NE‐CO; however, comprehensive evaluation of upstream and downstream signaling pathways and direct measurements of oxidative products, such as ROS and MDA, would strengthen the mechanistic interpretation. Future studies should therefore address the complete lipid composition, long‐term physicochemical stability, safety profile, and molecular pathways linking lipid‐mediated signaling, oxidative stress, inflammation, and renal fibrosis.

In conclusion, NE‐CO effectively attenuated obesity‐induced systemic and renal alterations, improving metabolic parameters, renal structure and function, and modulating the antioxidant and inflammatory responses while attenuating fibrotic changes. Importantly, the nanoemulsion demonstrated superior efficacy compared with free oil, likely due to enhanced bioavailability and reduced required dosage. These findings support NE‐CO as a promising therapeutic strategy for obesity‐associated kidney disease.

## Author Contributions

Conceptualization and study design: Priscila G. Pereira, Luciana L. Alves, and Bianca T. Ciambarella. Methodology: Priscila G. Pereira, Luciana L. Alves, Kíssila Rabelo, Ana Lúcia R. Nascimento, Alan Cesar N. Moraes, Andressa Bernardi, Fernanda V. Guimarães, Miguel P. Campos, Letícia Cruz, Denia M. S. Valladão. Writing first manuscript: Priscila G. Pereira and Luciana L. Alves. Editing: Bianca T. Ciambarella, Kíssila Rabelo, Cherley B. V. Andrade; Jemima F. R. da Silva, Andressa Bernardi, Stela R. Ferrarini. Reviewing: All authors and Jorge J. de Carvalho. Funding support: Jorge J. de Carvalho.

## Ethic Statement

All experimental procedures involving animals were approved by the Committee for Ethics in Animal Experimentation of the University of Rio de Janeiro State (n° 048/2022).

## Conflicts of Interest

The authors declare no conflicts of interest.

## Data Availability

Data available upon reasonable request.

## References

[1] L. O. Pereira , R. P. Francischi , and A. H. Lancha, Jr. , “Obesidade: Hábitos Nutricionais, Sedentarismo E Resistência À Insulina,” Arquivos Brasileiros de Endocrinologia and Metabologia 47, no. 2 (2003): 111–127, 10.1590/S0004-27302003000200003.

[2] World Obesity Federation . World Obesity Atlas (World Obesity Federation; 2023, 2023).

[3] C. Wickman and H. Kramer , “Obesity and Kidney Disease: Potential Mechanisms,” Seminars in Nephrology 33, no. 1 (2013): 14–22, 10.1016/j.semnephrol.2012.12.006. 23374890

[4] A. P. J. de Vries , P. Ruggenenti , X. Z. Ruan , et al., “Fatty Kidney: Emerging Role of Ectopic Lipid in Obesity‐Related Renal Disease,” Lancet Diabetes and Endocrinology 2, no. 5 (2014): 417–426, 10.1016/S2213-8587(14)70065-8. 24795255

[5] C. P. Kovesdy , S. L. Furth , and C. Zoccali , World Kidney Day Steering Committee ., “Obesity and Kidney Disease: Hidden Consequences of the Epidemic,” Journal of Nephrology 30, no. 1 (2017): 1–10, 10.1007/s40620-017-0377-y. 28214961

[6] T. Yamamoto , Y. Takabatake , A. Takahashi , et al., “High‐Fat Diet‐Induced Lysosomal Dysfunction and Impaired Autophagic Flux Contribute to Lipotoxicity in the Kidney,” Journal of the American Society of Nephrology 28, no. 5 (2017): 1534–1551, 10.1681/ASN.2016070731. PMC5407727 27932476

[7] S. Priyamvada , M. Priyadarshini , N. A. Arivarasu , et al., “Studies on the Protective Effect of Dietary Fish Oil on Gentamicin‐Induced Nephrotoxicity and Oxidative Damage in Rat Kidney,” Prostaglandins, Leukotrienes and Essential Fatty Acids 78, no. 6 (2008): 369–381, 10.1016/j.plefa.2008.04.008. 18556188

[8] A. Molfino , M. I. Amabile , M. Monti , and M. Muscaritoli , “Omega‐3 Polyunsaturated Fatty Acids in Critical Illness: Anti‐Inflammatory, Proresolving, or Both?,” Oxidative Medicine and Cellular Longevity 2017 (2017): 5987082, 10.1155/2017/5987082. PMC5488236 28694914

[9] H. Muramatsu , N. Akimoto , M. Hashimoto , K. Sugibayashi , and M. Katakura , “Influence of Polyunsaturated Fatty Acid Intake on Kidney Functions of Rats With Chronic Renal Failure,” Marine Drugs 19, no. 12 (2021): 692, 10.3390/md19120692. PMC8706431 34940691

[10] G. Ryeo‐Eun , H. Kyung‐A , P. Geon‐Tae , et al., “Effects of Microalgal Polyunsaturated Fatty Acid Oil on Body Weight and Lipid Accumulation in the Liver of C57BL/6 Mice Fed a High‐Fat Diet,” Journal of Biomedical Research 30, no. 3 (2016): 234–242, 10.7555/JBR.30.2016K0004. PMC4885172 27533934

[11] Y. Panahi , S. Dashti‐Khavidaki , F. Farnood , H. Noshad , M. Lotfi , and A. Gharekhani , “Therapeutic Effects of omega‐3 Fatty Acids on Chronic Kidney Disease‐Associated Pruritus: A Literature Review,” Advanced Pharmaceutical Bulletin 6, no. 4 (2016): 509–514, 10.15171/apb.2016.064. PMC5241408 28101457

[12] M. Fukushima , Y. Takayama , T. Habaguchi , and M. Nakano , “Comparative Hypocholesterolemic Effects of Capybara (*Hydrochoerus hydrochaeris dabbenei*) Oil, Horse Oil, and Sardine Oil in Cholesterol‐Fed Rats,” Lipids 32, no. 4 (1997): 391–395, 10.1007/s11745-997-0050-z. 9113627

[13] P. C. Marinho , A. B. Vieira , P. G. Pereira , et al., “Capybara Oil Improves Hepatic Mitochondrial Dysfunction, Steatosis, and Inflammation in a Murine Model of Nonalcoholic Fatty Liver Disease,” Evidence‐Based Complementary and Alternative Medicine 2018 (2018): 4956079, 10.1155/2018/4956079. PMC5949171 29853957

[14] L. Lontro Alves , P. Gomes Pereira , B. Torres Ciambarella , et al., “Beneficial Effects of Capybara Oil Supplementation on Steatosis and Liver Apoptosis in Obese Mice,” Journal of Obesity 2024 (2024): 7204607, 10.1155/2024/7204607. PMC11147678 38831961

[15] P. G. Pereira , L. L. Alves , B. T. Ciambarella , et al., “Capybara Oil Improves Renal Pathophysiology and Inflammation in Obese Mice,” Nutrients 15, no. 13 (2023): 2925, 10.3390/nu15132925. PMC10346293 37447251

[16] V. M. Esquerdo , G. L. Dotto , and L. A. A. Pinto , “Preparation of Nanoemulsions Containing Unsaturated Fatty Acid Concentrate‐Chitosan Capsules,” Journal of Colloid and Interface Science 445 (2015): 137–142, 10.1016/j.jcis.2014.12.094. 25617613

[17] D. de Sá Coutinho , J. Pires , H. Gomes , et al., “Pequi (Caryocar Brasiliense Cambess)‐Loaded Nanoemulsion, Orally Delivered, Modulates Inflammation in LPS‐Induced Acute Lung Injury in Mice,” Pharmaceutics 12 (2020): 1075, 10.3390/pharmaceutics12111075. PMC7696187 33187057

[18] E. Jäger , C. G. Venturini , F. S. Poletto , et al., “Sustained Release From Lipid‐Core Nanocapsules by Varying the Core Viscosity and the Particle Surface Area,” Journal of Biomedical Nanotechnology 5, no. 1 (2009): 130–140, 10.1166/jbn.2009.1004. 20055116

[19] M. C. Milinsk , M. Matsushita , J. V. Visentainer , et al., “Influência Do Método De Esterificação Na Quantificação De Ácidos Graxos Em Óleo De Oliva,” Semina: Ciências Exatas e Tecnológicas 32, no. 2 (2011): 139–150, 10.5433/1679-0375.2011v32n2p139.

[20] American Oil Chemists' Society ., Official Methods and Recommended Practices of the American Oil Chemists' Society (AOCS Press, 2004). 5th ed.

[21] A. L. Raiser , M. P. R. Torres , E. B. Ribeiro , and D. M. S. Valladão , “Characterization of Munguba Oil Obtained by Ultrasound,” Ciência Rural 50, no. 12 (2020): e20191028, 10.1590/0103-8478cr20191028.

[22] R. Geetanjali , V. Sreejit , P. Sandip , and R. Preetha , “Preparation of Aloe Vera Mucilage‐Ethyl Vanillin Nano‐Emulsion and its Characterization,” Materials Today: Proceedings 43 (2021): 3766–3773, 10.1016/j.matpr.2020.10.990.

[23] P. G. Reeves , F. H. Nielsen , and G. C. Fahey, Jr. , “AIN‐93 Purified Diets for Laboratory Rodents: Final Report of the American Institute of Nutrition Ad Hoc Writing Committee on the Reformulation of the AIN‐76A Rodent Diet,” Journal of Nutrition 123, no. 11 (1993): 1939–1951, 10.1093/jn/123.11.1939. 8229312

[24] E. Kennedy‐Feitosa , R. T. Okuro , V. Pinho Ribeiro , et al., “Eucalyptol Attenuates Cigarette Smoke‐Induced Acute Lung Inflammation and Oxidative Stress in the Mouse,” Pulmonary Pharmacology and Therapeutics 41 (2016): 11–18, 10.1016/j.pupt.2016.09.004. 27599597

[25] M. Winzell and B. Ahrén , “The High‐Fat Diet–Fed Mouse,” supplement, Diabetes 53, no. Suppl 3 (2004): S215–S219, 10.2337/diabetes.53.suppl_3.s215. 15561913

[26] O. C. Farokhzad and R. Langer , “Impact of Nanotechnology on Drug Delivery,” ACS Nano 3, no. 1 (2009): 16–20, 10.1021/nn900002m. 19206243

[27] N. Deji , S. Kume , S. Araki , et al., “Structural and Functional Changes in the Kidneys of High‐Fat Diet‐Induced Obese Mice,” American Journal of Physiology‐Renal Physiology 296, no. 1 (2009): F118–F126, 10.1152/ajprenal.00110.2008. 18971213

[28] N. Kambham , G. S. Markowitz , A. M. Valeri , J. Lin , and V. D. D'Agati , “Obesity‐Related Glomerulopathy: An Emerging Epidemic,” Kidney International 59, no. 4 (2001): 1498–1509, 10.1046/j.1523-1755.2001.0590041498.x. 11260414

[29] R. Braddock , C. Siman , K. Hamilton , H. Devlin , H. Garland , and C. Sibley , “Gamma‐Linoleic Acid and Ascorbic Acid Ameliorate the Effects of Experimental Diabetes on Electrolyte and Bone Homeostasis in Pregnant Rats,” Journal of Endocrinology 173, no. 2 (2002): 273–284, 10.1677/joe.0.1730273. 12010635

[30] H. J. Mattix and K. F. Badr , “Arachidonic Acid Metabolites and the Kidney.” in The Kidney, eds. B. M. Brenner (WB Saunders, 2000). 6th ed., 774–779.

[31] L. N. Masi , A. R. Martins , J. C. R. Neto , et al., “Sunflower Oil Supplementation has Proinflammatory Effects and Does not Reverse Insulin Resistance in Obesity Induced by High‐Fat Diet in C57BL/6 Mice,” Journal of Biomedicine and Biotechnology 2012 (2012): 1–9, 10.1155/2012/945131. PMC3441046 22988427

[32] L. Landsberg , L. J. Aronne , L. J. Beilin , et al., “Obesity‐Related Hypertension: Pathogenesis, Cardiovascular Risk, and Treatment: A Position Paper of the Obesity Society and the American Society of Hypertension,” Journal of Clinical Hypertension 15, no. 1 (2013): 14–33, 10.1111/jch.12049. PMC8108268 23282121

[33] A. P. Simopoulos , “Omega‐6/omega‐3 Essential Fatty Acids: Biological Effects,” World Review of Nutrition and Dietetics 99 (2009): 1–16, 10.1159/000192755. 19136835

[34] E. Katsoulieris , J. G. Mabley , M. Samai , M. A. Sharpe , I. C. Green , and P. K. Chatterjee , “Lipotoxicity in Renal Proximal Tubular Cells: Relationship Between Endoplasmic Reticulum Stress and Oxidative Stress Pathways,” Free Radical Biology and Medicine 48, no. 12 (2010): 1654–1662, 10.1016/j.freeradbiomed.2010.03.021. 20363316

[35] J. L. Gorriz and A. Martinez‐Castelao , “Proteinuria: Detection and Role in Native Renal Disease Progression,” Transplantation Reviews 26, no. 1 (2012): 3–13, 10.1016/j.trre.2011.10.002. 22137726

[36] P. Garg , “A Review of Podocyte Biology,” supplement, American Journal of Nephrology 47, no. Suppl 1 (2018): 3–13, 10.1159/000481633. 29852492

[37] I. Pérez‐Torres , V. Castrejón‐Téllez , M. E. Soto , M. E. Rubio‐Ruiz , L. Manzano‐Pech , and V. Guarner‐Lans , “Oxidative Stress, Plant Natural Antioxidants, and Obesity,” International Journal of Molecular Sciences 22, no. 4 (2021): 1786, 10.3390/ijms22041786. PMC7916866 33670130

[38] J. C. Arroyave‐Ospina , Z. Wu , Y. Geng , and H. Moshage , “Role of Oxidative Stress in the Pathogenesis of Non‐Alcoholic Fatty Liver Disease: Implications for Prevention and Therapy,” Antioxidants 10, no. 2 (2021): 174, 10.3390/antiox10020174. PMC7911109 33530432

[39] B. P. Yu , “Cellular Defenses Against Damage From Reactive Oxygen Species,” Physiological Reviews 74, no. 1 (1994): 139–162, 10.1152/physrev.1994.74.1.139. 8295932

[40] S. Y. Lee and M. E. Choi , “Urinary Biomarkers for Early Diabetic Nephropathy: Beyond Albuminuria,” Pediatric Nephrology 30, no. 7 (2015): 1063–1075, 10.1007/s00467-014-2888-2. PMC4305495 25060761

[41] J. E. Kim , M. H. Lee , D. H. Nam , et al., “Celastrol, an NF‐κB Inhibitor, Improves Insulin Resistance and Attenuates Renal Injury in db/db Mice,” PLoS One 8, no. 4 (2013): e62068, 10.1371/journal.pone.0062068. PMC3637455 23637966

[42] S. Ogawa , H. Kobori , N. Ohashi , et al., “Angiotensin II Type 1 Receptor Blockers Reduce Urinary Angiotensinogen Excretion and the Levels of Urinary Markers of Oxidative Stress and Inflammation in Patients With Type 2 Diabetic Nephropathy,” Biomarker Insights 4 (2009): BMI.S2733, 10.4137/BMI.S2733. PMC2716676 19652765

[43] E. P. Böttinger , “TGF‐β in Renal Injury and Disease,” Seminars in Nephrology 27, no. 3 (2007): 309–320, 10.1016/j.semnephrol.2007.02.009. 17533008

[44] M. H. Cho , “Renal Fibrosis,” Korean Journal of Pediatrics 53, no. 7 (2010): 735–740, 10.3345/kjp.2010.53.7.735. PMC3004484 21189948

[45] T. Nagamoto , G. Eguchi , and D. C. Beebe , “Alpha‐Smooth Muscle Actin Expression in Cultured Lens Epithelial Cells,” Investigative Ophthalmology and Visual Science 41, no. 5 (2000): 1122–1129. 10752950

[46] J. L. Barnes and W. F. Glass, II , “Renal Interstitial Fibrosis: A Critical Evaluation of the Origin of Myofibroblasts,” Contributions to Nephrology 169 (2011): 73–93, 10.1159/000313946. 21252512

[47] W. Kriz , B. Kaissling , and M. Le Hir , “Epithelial‐Mesenchymal Transition (EMT) in Kidney Fibrosis: Fact or Fantasy,” Journal of Clinical Investigation 121, no. 2 (2011): 468–474, 10.1172/JCI44595. PMC3026733 21370523

[48] X. Palomer , J. Pizarro‐Delgado , E. Barroso , and M. Vázquez‐Carrera , “Palmitic and Oleic Acid: The Yin and Yang of Fatty Acids in Type 2 Diabetes Mellitus,” Trends in Endocrinology and Metabolism 29, no. 3 (2018): 178–190, 10.1016/j.tem.2017.11.009. 29290500

[49] P. C. Calder , “n‐3 Polyunsaturated Fatty Acids, Inflammation, and Inflammatory Diseases,” supplement, American Journal of Clinical Nutrition 83, no. 6 Suppl (2006): 1505S–1519S, 10.1093/ajcn/83.6.1505S. 16841861

[50] O. Izaola , D. de Luis , I. Sajoux , J. C. Domingo , and M. Vidal , “Inflammation and Obesity (Lipoinflammation),” Nutricion Hospitalaria 31, no. 6 (2015): 2352–2358, 10.3305/nh.2015.31.6.8829. 26040339

[51] R. Düsing , A. Struck , B. O. Göbel , B. Weisser , and H. Vetter , “Effects of n‐3 Fatty Acids on Renal Function and Renal Prostaglandin E Metabolism,” Kidney International 38, no. 2 (1990): 315–319, 10.1038/ki.1990.202. 2402124

[52] A. Friedman and S. Moe , “Review of the Effects of omega‐3 Supplementation in Dialysis Patients,” Clinical Journal of the American Society of Nephrology 1, no. 2 (2006): 182–192, 10.2215/CJN.00740805. 17699207

